# First long-term outcome data for the MicraVR™ transcatheter pacing system: data from the largest prospective German cohort

**DOI:** 10.1007/s00392-023-02286-1

**Published:** 2023-08-22

**Authors:** Arian Sultan, Cornelia Scheurlen, Jonas Wörmann, Jan-Hendrik van den Bruck, Karlo Filipovic, Susanne Erlhöfer, Sebastian Dittrich, Jan-Hendrik Schipper, Jakob Lüker, Jan-Malte Sinning, Dinh Quang Nguyen, Sören Fischer, Daniel Steven, Stefan Winter

**Affiliations:** 1 Department of Electrophysiology, Heart Center, University of Cologne, Cologne, Germany, Kerpener Str. 62, 50937 Cologne, Germany; 2 St. Vinzenz Hospital Cologne, Cologne, Germany

**Keywords:** Leadless pacer, Outside registry experience, Long-term follow-up, Indications, Complications

## Abstract

**Aims:**

The MicraVR™ transcatheter pacing system (TPS) has been implemented into clinical routine for several years. The primary recipients are patients in need for VVI pacing due to bradycardia in the setting of atrial fibrillation (AF). Implantation safety and acute success have been proven in controlled studies and registries.

So far only few long-term real-life data on TPS exist. We report indication, procedure and outcome data from two high-volume implanting German centers.

**Methods:**

Between 2016 and 2019, 188 (of 303) patients were included. During follow-up (FU), TPS interrogation was performed after 4 weeks and thereafter every 6 months.

**Results:**

Indication for TPS implantation in 159/188 (85%) patients was permanent or intermittent AV block III° in the setting of atrial fibrillation.

The mean procedure duration was 50 min [35.0–70.0]. The average acute values after system release were: thresholds: 0.5V [0.38–0.74]/0.24ms; R-wave sensing: 10.0mV [8.1–13.5]; impedance: 650 Ohm [550–783]; RV-pacing demand: 16.9% [0.9–75.9]; and battery status: 3.15 V [3.12–3.16]. During FU of 723.4 ± 597.9 days, neither pacemaker failure nor infections were reported.

Long-term FU revealed: thresholds: 0.5V [0.38–0.63]/0.24 ms; sensing: 12.3mV [8.9–17.2]; impedance: 570 Ohm [488–633]; RV-pacing demand: 87.1% [29.5–98.6]; and battery status 3.02 V [3.0–3.1]. Forty-three patients died from not-device-related causes.

**Conclusion:**

This to date largest German long-term dataset for MicraVR™ TPS implantation revealed stable device parameter. Foremost, battery longevity seems to fulfill predicted values despite a significant increase in RV-pacing demand over time and even in patients with consecutive AV-node ablation. Of note, no infections or system failure were observed.

**Graphical abstract:**

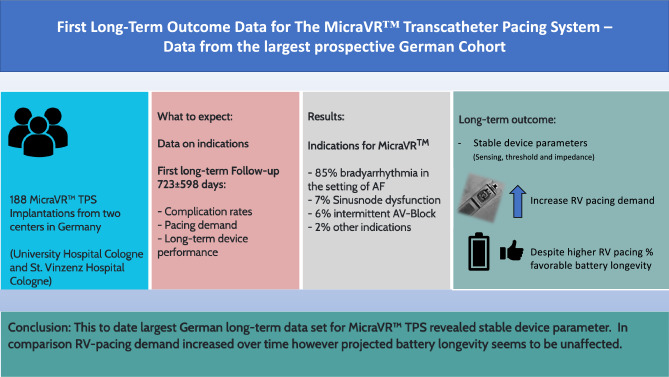

**Supplementary Information:**

The online version contains supplementary material available at 10.1007/s00392-023-02286-1.

## What's new?

- To date this cohort is the largest German cohort experience in the MicraVR™ transcatheter pacing system.
- In long-term follow up pacing threshold, r-wave amplitude, impedance, and battery show stable values. In comparison RV-pacing demand increased over time however projected battery longevity seems to be unaffected.

## Introduction

The MicraVR™ transcatheter pacing system (TPS) has been implemented into clinical routine for several years. As opposed to conventional transvenous pacemaker implantation, the use of leadless TPS device omits lead- and access-related complications such as hematoma, lead fracture, pneumothorax, twiddler syndrome, pocket infections and venous thrombosis [1, 2]. The primary recipients of MicraVR™ are patients in need for VVI pacing due to bradycardia in the setting of atrial fibrillation (AF). Feasibility, implantation safety and acute success have been proven in the setting of controlled studies. Some 12- and up to 24-month follow-up (FU) data from this controlled study population and from a post-approval registry are available reporting stable device values with only few complications [3–7] and no device-related infections. However, true long-term FU data for MicraVR in a real-world setting assessing not only device performance but foremost battery longevity, possible complications and indications MicraVR is sparse. The predicted battery longevity in TPS is specified 8–13 years [8].

Therefore, we sought to report long-term outcome data from the, to our knowledge, largest German cohort of MicraVR TPS recipients, not only focusing on conventional device values but RV-pacing demand battery longevity and RV function. Furthermore, we provide data on MicraVR indications and feasibility outside the typical VVI indication for bradycardia in the setting of persistent or permanent AF.

## Methods

### Study population

Between January 2016 and December 2019, 188 patients underwent successful TPS implantation at the University Heart Center of Cologne and the St. Vinzenz Hospital in Cologne. Overall, 303 MicraVR TPS implantations were performed in these high-volume centers since MicraVR launch. Inclusion criteria were TPS implantation, age > 18 years and having given written informed consent. Recipients consisted mainly of patients with a guideline-based class I or II indication for demand ventricular pacing (VVI pacing) [9].

The study complied with the Declaration of Helsinki. All included participants signed informed consent to the procedure and the general data processing and analysis.

### Implantation procedure

The single-chamber TPS pacing device (*Medtronic, Minneapolis, MN, USA*) is a self-contained capsule of 0.8 cm^3^ and 2.0 g. The TPS features adaptive rate response and an automated pacing-capture threshold management. Furthermore, it is unrestrictedly MRI conditionally safe for full-body 1.5-T and 3.0-T. The device is embedded in a steerable catheter, which is also used for the device application. Catheter and application details using a femoral vein access as well as implantation specifics have been described previously [10].

All procedures were performed in deep sedation using weight-adjusted propofol, midazolam and fentanyl. A continuous oxygen insufflation and airway protection were established. As a precaution and visual guidance, a temporary RV-pacing lead (*Supreme™, St. Jude Medical, Minnesota, USA*) was placed into the RV to obtain optimal implantation conditions in the case of an intermittent AV block. As opposed to using an RV-pacing catheter, the use of contrast medium to perform a RV angiography might be equally helpful to understand patients’ anatomy and was also performed in some cases.

After MicraVR deployment into the right ventricular (RV) myocardium, the fixation of nitinol tines was stressed by tug testing.

Fluoroscopy proof of at least two firmly anchored tines was obligatory before releasing the MicraVR device from the application catheter. Device interrogation was performed before and after tug testing; if device values declined, the TPS was retracted, and a new position was approached.

A minimum R-wave sensing of 6 mV and a pacing threshold of < 1 V/0.24 ms were strived during implantation. After the final deployment of the TPS a repeat device interrogation was performed immediately and at discharge. For the sheet-removal and puncture closure, a Z-suture was applied. At the end of the procedure and 2 h after, pericardial effusion was excluded using an echocardiogram.

At the following day, a device interrogation was performed in our device clinic. The evaluated measurements at this interrogation were defined as the implantation values.

### Follow-up

At first follow-up (FU) after 4 weeks, all patients were seen at the initially implanting center. Consecutive FUs every 6 months were either performed at implanting centers or associated cardiologist. Evaluated parameters during FU consisted of device values such as pacing threshold, R-wave amplitude, impedance, battery status, RV-pacing demand as well as patients’ clinical status. These electrical parameters were analyzed independently of each other with regard to sensing, pacing threshold and impedance values. Furthermore, battery performance and RV-pacing demand were analyzed and put into perspective. Also, groins were checked for belated hematoma or other complications.

The comparison of the device values in this analysis was performed between the implantation values and the measured values at the latest follow-up of each patient.

### Statistical analysis

Continuous data were displayed as mean and standard deviation and categorical variables as counts and percentages. For skewed data, median and interquartile range were used. Statistical significance was evaluated by Student’s *t* test. A *p* value < 0.05 was considered statistically significant.

## Results

### Baseline characteristics

In total, 188 patients (108 men, 57%) were included in this retrospective analysis. In all procedures, TPS was implanted successfully. The average age was 79.9 ± 8.6 years. In 10% of these implantations, a pre-existing device was explanted due to, e.g., infection, thrombosis or lead/device malfunction. The most frequent comorbidity was atrial fibrillation (86%). In 20% of the included patients, an AV-node ablation was performed consecutively due to atrial fibrillation. The baseline characteristics and other comorbidities are shown in Table 1.

**Table 1:** Baseline characteristics

Baseline characteristics	
Patients, *n*	188
Age [years]	79.7 ± 8.6
Male gender, *n* (%)	108 (57%)
Pre-existing device, %	10%
*Comorbidities*	
Persistent/permanent AF, %	86%
AV-node ablation, %	20%
Arterial hypertension, %	70%
Diabetes mellitus type II, %	23%
Chronic renal insufficiency, %	46%
Prior stroke, %	18%
CAD, %	35%
Heart failure, %	26%
Median LV-EF [%]	55
COPD, %	8%
*Pacing indication*	
Bradyarrhythmia with AF, *n* (%)	159 (85%)
Sinus node dysfunction, *n* (%)	14 (7%)
AV block, *n* (%)	12 (6%)
Others, *n* (%)	3 (2%)

Continuous data are summarized as means ± standard deviation or median [interquartile range]; categorical data are presented as number (percent)

*AF* atrial fibrillation, *CAD* coronary artery disease, *COPD* chronic obstructive pulmonary disease, *EF* ejection fraction, *LV* left ventricle, *n* number

### Indications

**Fig. 1:**
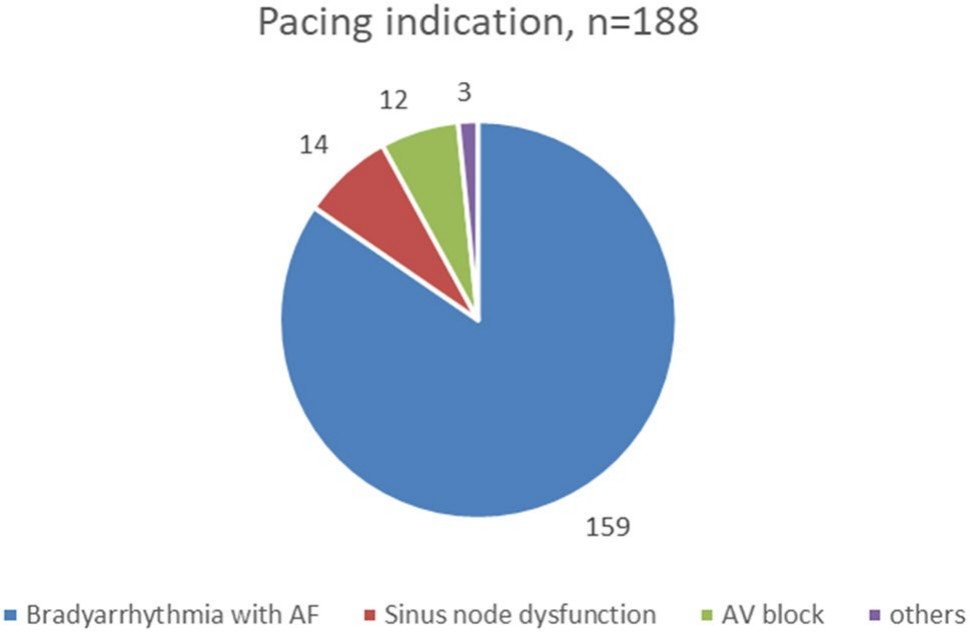
Pacing indication. Pie chart displays distribution of different pacing indication of the overall TPS recipients (*n* = 188) cohort. *AF* atrial fibrillation

The vast majority of TPS recipients (159, 85%) fulfilled a typical VVI pacing indication due to symptomatic bradyarrhythmia in the setting of persistent or permanent atrial fibrillation. Besides this indication, there were some cases with sinus node dysfunction (14 cases, 7%), intermittent higher AV blockage in setting of sinus rhythm (12 cases, 6%) and other indications (mainly anatomical obstacles) (3 cases, 2%) [11]. The pacing indications are shown in Fig. 1. Since data on battery longevity were sparse at that time, in most patients undergoing TPS implantation a low RV-pacing demand was expected.

### Procedural characteristics

The mean procedure duration was 50.0 min [35.0–70.0]. The mean fluoroscopy dose was 1953.0 µGy × m^2^ [954.0–3401.0] and the mean fluoroscopy time 8.0 min [5.23–11.3]. The number of attached splines to the RV myocardium, proven in two different fluoroscopy angulations, was in median three tines [2, 3]. Only in a few cases (*n* = 21, 11%), a re-positioning of the device due to insufficient tine-tissue engagement was necessary. In five cases, over 10 deployment attempts were conducted until a stable TPS position with acceptable device values was achieved. The procedural characteristics are summarized in Table 2.

**Table 2:** Procedural characteristics and follow-up data

Procedural characteristics	
Procedure time [min]	50.0 [35.0–70.0]
Fluoroscopy dose [µGy × m^2^]	1953.0 [954.0–3401.0]
Fluoroscopy time [min]	8.0 [5.23–11.3]
Splines, n	3 [2–3]
Intraprocedural complications, *n* (%)	1 (0.5%)
Postprocedural complications, *n* (%)	1 (0.5%)
*Follow-up data*	
Follow-up period [days]	723.4 ± 597.9
Adjusted follow-up period* [days]	810.5 ± 596.0
Death during follow-up, *n* (%)	43 (23%)
Second MicraVR implantation, *n* (%)	2 (1%)
Device change, *n* (%)	3 (2%)

*n* number

^*^Adjusted follow-up period: deceased patients and patients with second MicraVR implantation and device change during follow-up period were excluded

Mean acute values after system release were: RV-pacing thresholds of 0.5 V [0.38–0.75]/0.24 ms, R-wave amplitude of 10.0 mV [7.1–13.5] and a device impedance of 650 Ohm [550–783] (Table 3). RV-pacing demand was initial 16.9% [0.9–75.9] and battery status was 3.15 V [3.12–3.16] (Table 3).

**Table 3:** Median measurements

Measurements	Implantation	Max. follow-up	*p* value
Pacing threshold [V at 0.24 ms]	0.5 [0.38–0.75]	0.5 [0.38–0.63]	0.78
R-wave amplitude [mV]	10.0 [7.1–13.5]	12.3 [8.9–17.2]	< 0.001*
Impedance [ohm]	650 [550–783]	570 [488–633]	< 0.001*
RV pacing [%]	16.9 [0.9–75.9]	87.1 [29.5–98.6]	< 0.001*
Battery [V]	3.15 [3.12–3.16]	3.02 [3.0–3.1]	< 0.001*

*mV* millivolt, *RV* right ventricular, *V* Volt

*Statistically significant

### Complication and device safety

In our cohort, two periprocedural complications (1.1%) occurred: one patient developed an intraprocedural pericardial effusion and another patient a postprocedural pericardial effusion. Both complications could be handled by pericardiocentesis. No device failure was reported acutely or until patient discharge. Also, no groin complication occurred. In two cases, a transient complete AV block III° with severe bradycardia occurred while positioning the delivery sheet into the RV. Therefore, a temporary RV-pacing lead was placed to obtain optimal implantation conditions. During the complete FU period, neither pacemaker failure, nor infections or further complications were reported.

### Follow-up

The average FU time was 723.4 ± 597.9 days. During FU, 43 (23%) patients died from not-device-related causes, mainly age related. In addition, three CRT implantations due to worsening left ventricular function and two second MicraVR implantations were performed during FU. In the case of adjusting the FU time by exclusion of the deceased patients and patients with a device change or second MicraVR implantation during FU, the mean follow-up time was 810.5 ± 596.0 days.

**Fig. 2:**
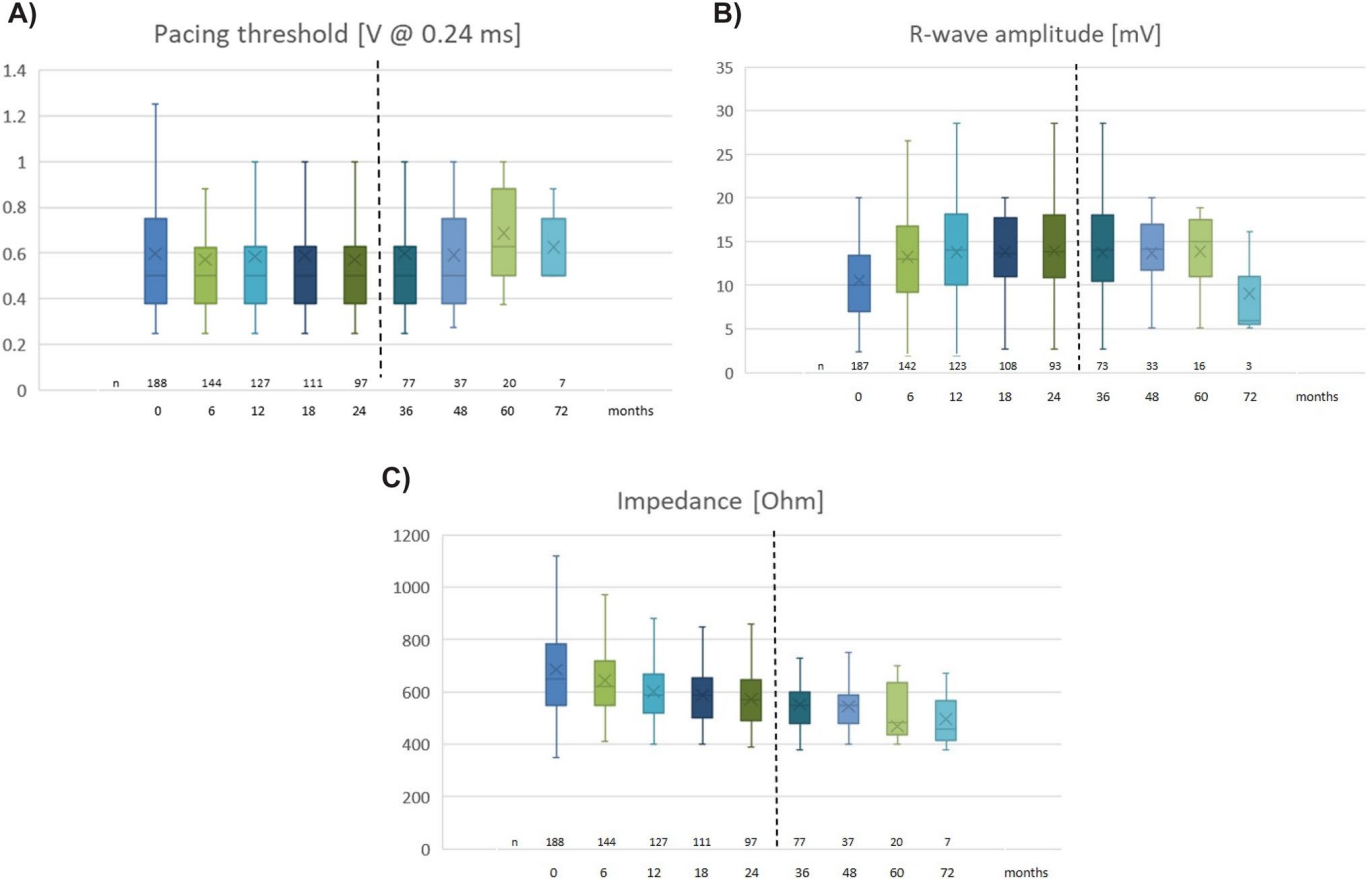
Electrical parameters during follow-up in months. **A** Pacing threshold [V @ 0.24 ms]. Whisker’s plot displays pacing threshold [V @ 0.24 ms] during follow-up in months. Starting point (0 month) shows the respective electrical parameter at implantation. *n* numbers at risk. **B** R-wave amplitude [mV] during follow-up in months. Whisker’s plot displays R-wave amplitude [mV] during follow-up in months. Starting point (0 month) shows the respective electrical parameter at implantation. Data measured if own heart rhythm was available. *n* numbers at risk. **C** Impedance [Ohm] during follow-up in months. Whisker’s plot displays pacing impedance [Ohm] during follow-up in months. Starting point (0 month) shows the respective electrical parameter at implantation. *n* numbers at risk

In comparison, a few significant changes from acute to long-term measurements regarding TPS values and performance were detectable. Pacing threshold was comparable between implantation (0.5 V [0.38–0.75]/0.24ms) and max. FU (0.5V [0.38–0.63]/0.24 ms, *p* = 0.78) (Fig. 2A). At implantation, 88.3% had a pacing threshold ≤ 1 V and 96.8% ≤ 1.5 V. The R-wave amplitude (Fig. 2B) as well as the impedance (Fig. 2C) improved statistically significant between time of implantation (R-wave amplitude: 10.0 mV [7.1–13.5]; impedance: 650 Ohm [550–783]) and max. FU (max. FU: R-wave amplitude: 12.3 mV [8.9–17.2], *p* < 0.001; impedance: 570 Ohm [488–633], *p* < 0.001).

**Fig. 3:**
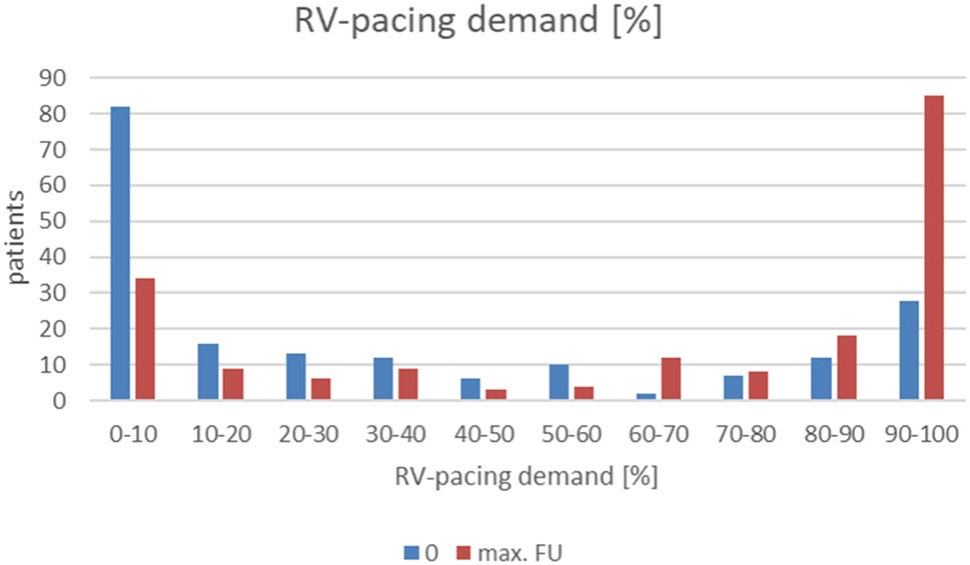
RV-pacing demand [%] during follow-up. Bar chart displays RV-pacing demand [%], divided into categories with 0–10% RV-pacing demand, 10–20%, 20–30%, …, 90–100% RV-pacing demand, during follow-up. Blue bar chart represents RV-pacing demand at implantation; red bar chart represents RV-pacing demand at max. FU. *FU* follow-up

Of note, the RV-pacing demand (Fig. 3) increased statistically significant during the FU time (implantation: 16.9% [0.9–75.9]; max. FU: 87.1% [29.5–98.6], *p* < 0.001).

**Fig. 4:**
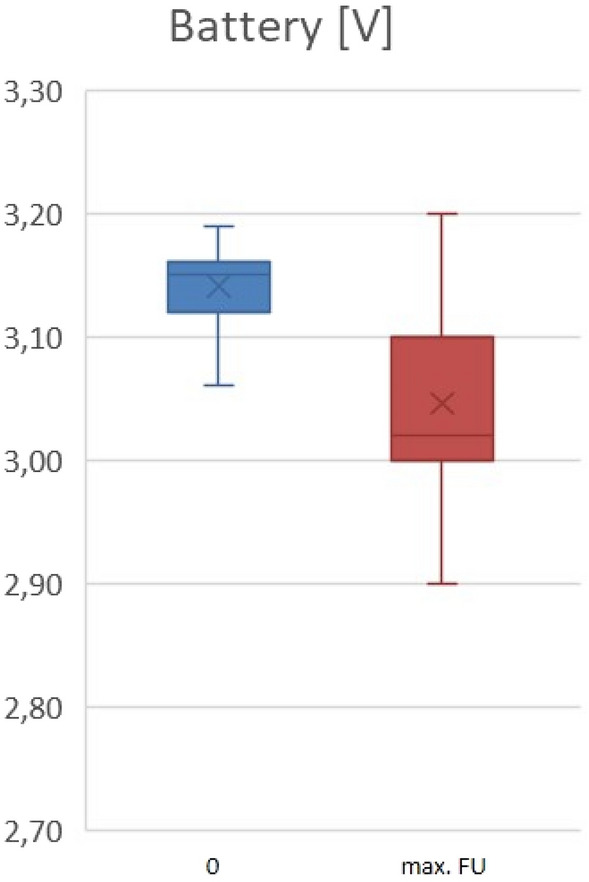
Battery [V] during follow-up. Whisker’s plot displays battery status during follow-up. Starting point (0 month) shows battery status at first device interrogation following implantation. FU: follow-up

The battery consumption decreased over the time expectedly (implantation: 3.15 V [3.12–3.16]; max. FU: 3.02 V [3.0–3.1], *p* < 0.001) without any signs of early battery depletion (Fig. 4). FU data are displayed in Table 3**.**

Furthermore, the long-term course of patients with initial high pacing threshold, defined as pacing threshold > 1.25 V/0.24 ms, and initial low R-wave amplitude, defined as R-wave amplitude < 6 mV, was evaluated. Overall, initial high pacing threshold and low R-wave amplitude remained stable or even improved during follow-up (Fig. S1).

In one patient, a transient impairment of TPS values was detectable acutely after MRI for suspected stroke [12]. The MRI was performed 5 months after TPS implantation. All values went back to initial values before MRI after 48 h. In this particular patient, implanting threshold and pacing values were not optimal but were accepted after several positioning attempts. To our knowledge, this was the only patient who did undergo MRI after TPS implantation.

## Discussion

### Main findings

These data were obtained in a real-world setting outside the so far published registry data and to our knowledge represent the largest German cohort experience on leadless MicraVR TPS implantation and the so far longest FU for TPS implantation. The main findings of this analysis are as follows:

1. A very high acute implantation success rate of 100% was achieved in both centers with no significant complications (1.1%).
2. No infections or system failure occurred acutely or during long-term FU.
3. Device interrogation showed acceptable values as well in long-term FU with stable battery status despite a significant increase in RV-pacing demand.

### Study population

As compared to the registry data, the published post-approval registry data and the 12-month TPS data [3–5], this German cohort’s data consist of mainly older (average age: 79.9 ± 8.6 years) de novo pacemaker implantation patients with a very long FU. Only 10% had a previous transvenous system as opposed to 14.5% in the post-approval registry [5]. Also, the proportion of patients suffering from AF and therefore undergoing TPS implantation for a typical VVI pacing indication is slightly higher in our cohort as compared to previous published data (85% vs. 66.9% [5] vs. 72.6% [3]). Regarding further patients’ demographics, no major differences are detectable.

### Non-VVI pacing indication

In this cohort, a noteworthy number of patients (15%) received a TPS outside the typical VVI pacing indication. Aside from anatomical obstacles precluding a conventional PM system, the majority of these patients suffered from several comorbidities, in particular diabetes mellitus type II und renal failure, partly end-stage renal failure with dialysis indication. The estimated prevalence of CIED for patients with end-stage renal failure is roughly 10.5% [13].

In this context, several studies highlight the significantly altered risk of potential CIED infection and venous stenosis in the peculiar set of patients with end-stage renal failure undergoing dialysis [14–16]. Therefore, a non-transvenous system like the TPS offers an actual alternative to possibly avoid fatal complications in the setting of CIED and end-stage renal failure.

Despite significant higher initial costs implanting a TPS system, implantation of the latter might still be justified in selected patients at high risk of infection or other obstacles. Data show, if lead-associated complications occur, hospitalization and consecutive treatment costs are high and significantly exceed the cost of a TPS systems [17].

Besides the typical VVI pacing indication for TPS implantation, since 2020 MicraAV was introduced in the German market with atrioventricular synchrony. In this analysis, no MicraAV was included. However, in future indication for TPS implantation could be extended and long-term data on TPS are lacking until now.

### Procedural obstacles and lessons

**Fig. 5:**
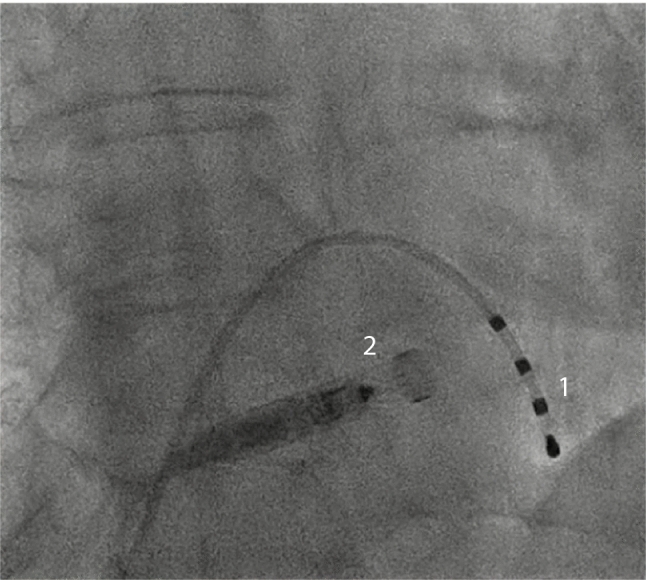
Fluoroscopy of TPS implantation. Transfemoral-placed quadripolar pacing lead (1). It may function as 1. Back-up pacing in the case of intermittent AVB III° and 2. A visual marker and therefore potentially facilitates TPS (2) introduction into the RV (tricuspid valve passage). *RV* right ventricle, *TPS* transcatheter pacing system

As reported, in two patients a transient complete AV block III° with a severe bradycardia occurred during placement of the TPS sheet in the RV. After the first case of complete AV block, standard placement of a transient pacing lead in the inferior vena cava was incorporated into our implantation setup. With this precaution, the second complete AV block was countered immediately and TPS implantation proceeded without a delay or longer period of severe bradycardia. Furthermore, the transfemoral-placed pacing lead (*Supreme™, St. Jude Medical, Minnesota, USA*) provides a visual guidance into the RV and therefore potentially facilitates TPS introduction into the RV (Fig. 5).

It is noteworthy that all implanting physicians had previous experience in electrophysiological studies, catheter ablation and CIED implantation. Furthermore, all implanters were trained by Medtronic and observed by a Medtronic representative during the first 20 cases, which surely warrants high implantation safety and high implantation success rates.

### Complications and device safety

As compared to previously published data [3–5], in our cohort only two periprocedural complications (1.1%) occurred ([3] 4%, [4] 4%, [5] 1.5%). In long-term FU, no system failure or infection was reported. Our data clearly demonstrate that TPS implantation is not only safe acutely but also during long-term FU in a real-world setting.

### Follow-up

Regarding implantation techniques, it seems to be crucial to obtain favorable acute TPS parameters to also achieve good long-term results and a stable TPS position. As already shown by Roberts et al., during the FU device interrogation showed acceptable, stable electrical parameters such as threshold, R-wave amplitude and impedance [5]. In our analysis, besides these stable electrical parameters, a significant increase in RV-pacing demand could be detected assumingly due to intensified β-blocker dosage or progression to higher-degree AV block. This finding is noteworthy because initially patients with an expected low RV-pacing demand were elected for TPS implantation. As already mentioned, the predicted battery longevity in TPS is specified 8–13 years [8]. Of note, despite this significant increase in RV-pacing demand, the battery status stayed stable during FU. Therefore, the projected battery longevity of TPS will be fulfilled and potentially even exceeded.

### Limitations

In accordance with previous studies, a control group with conventional devices is lacking. Also, the vast majority of patients underwent a de novo TPS implantation. The presented data are gathered from “only” two German centers on a heterogenic study population; however, these two centers reflect to date the largest cohort of TPS implantation with the so far longest follow-up in Germany. Also, the included patients reflect the possible recipients of a TPS outside registry data.

## Conclusion

This to date largest German cohort experience with a very long real-world FU shows that leadless TPS implantation is not only safe acutely but also during long-term FU fulfilling and potentially surpassing expected battery longevity. Furthermore, it should be considered as a safe and effective alternative for patients with comorbidities or anatomical obstacles who may not fulfill the typical VVI pacing indications providing stable long-term system parameters and possibly avoiding severe complications in the long run.

## Supplementary Information

Below is the link to the electronic supplementary material.

Supplementary file1 (DOCX 67 kb)
