# Supplementary material for: First long-term outcome data for the MicraVR™ transcatheter pacing system: data from the largest prospective German cohort
Source: Clin Res Cardiol. 2023 Aug 22;113(10):1443–50. doi: 10.1007/s00392-023-02286-1 (PMC11420340; doi:10.1007/s00392-023-02286-1)

# Supplement

**B**

**Figure S1: Long-term course of patients with initial high pacing threshold (A) and low R-wave amplitude (B)**

Dot charts display long-term course of patients with initial high pacing threshold (A), defined as pacing threshold >1.25V (n=7), and initial low R-wave amplitude (B), defined as R-wave amplitude <6mV (n=20). Starting point (0 month) show respective values at implantation.

**A) B)**


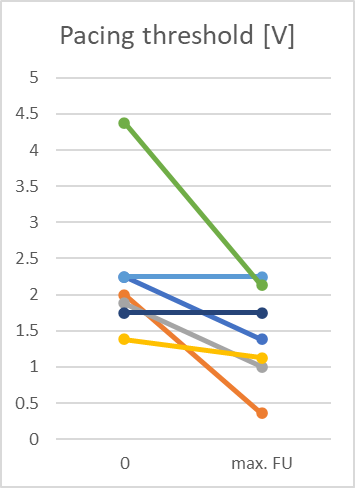

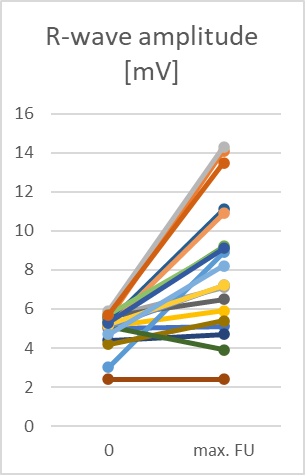

Supplement: Supplementary file 1 — Supplementary file1 (DOCX 67 kb) [file 392_2023_2286_MOESM1_ESM.docx]
